# Dietary Acid Load and Cardiometabolic Risk Factors—A Narrative Review

**DOI:** 10.3390/nu12113419

**Published:** 2020-11-07

**Authors:** Joanna Ostrowska, Justyna Janiszewska, Dorota Szostak-Węgierek

**Affiliations:** Department of Clinical Dietetics, Faculty of Health Sciences, Medical University of Warsaw, E Ciołka Str. 27, 01-445 Warsaw, Poland; jostrowska@wum.edu.pl (J.O.); dorota.szostak-wegierek@wum.edu.pl (D.S.-W.)

**Keywords:** dietary acid load, hypertension, insulin resistance, type 2 diabetes mellitus, lipid profile

## Abstract

The Western, diet rich in acidogenic foods (e.g., meat, fish and cheese) and low in alkaline foods (e.g., vegetables, fruits and legumes), is deemed to be a cause of endogenous acid production and elevated dietary acid load (DAL), which is a potential cause of metabolic acidosis. Multiple authors have suggested that such a dietary pattern increases the excretion of calcium and magnesium, as well as cortisol secretion. In addition, it is associated with decreased citrate excretion. All of these seem to increase blood pressure and insulin resistance and may contribute to the development of cardiometabolic disorders. However, there are inconsistencies in the results of the studies conducted. Therefore, this narrative literature review aims to present the outcomes of studies performed in recent years that investigated the association between DAL and the following cardiometabolic risk factors: blood pressure, hypertension, carbohydrate metabolism and lipid profile. Study outcomes are divided into (i) statistically significant positive association, (ii) statistically significant inverse association, and (iii) no statistically significant association.

## 1. Introduction

It is well established that diet may influence the acid–base balance in the body, which is becoming increasingly significant in human nutrition sciences. A diet rich in acidogenic foods (including meat, fish, cheese, rice and cereals) and low in alkaline foods (including fruits, vegetables, legumes, potatoes and red wine) seems to contribute to endogenous acid production and elevated dietary acid load (DAL), which impedes the maintenance of optimal body acid–base balance and is a potential cause of metabolic acidosis [1,2]. A diet rich in food that has a relatively high acid-forming potential is characteristic of the Western dietary pattern and may contribute to the development of cardiometabolic disorders (e.g., arterial hypertension, insulin resistance, diabetes) and cancer and seems to promote osteoporosis as well as renal calculosis [2,3,4,5,6,7,8,9]. The three main methods of calculating DAL are (i) potential renal net acid load (PRAL)—a negative value indicates a base-forming potential of the food consumed, while a positive value points to its acid-forming potential [1]; (ii) net endogenous acid production (NEAP), indicating a high intake of animal proteins [10]; (iii) net acid excretion (NAE, indicating the excess of dietary anions [11]. It has been suggested that PRAL is the most relevant measurement of DAL. It includes dietary protein intake and numerous micronutrients, such as potassium, magnesium, calcium and phosphorus, and takes into account the absorption rate of nutrients in the intestinal border [12]. 

Several studies have indicated significant relationships between the Western dietary pattern and the increased prevalence of cardiometabolic risk factors. However, the obtained results are frequently contradictory. Therefore, it is necessary to conduct a comprehensive overview of the studies in this field. In order to analyze this issue in more detail, studies from recent years were divided based on differences in the significance of the association between DAL and the analyzed cardiometabolic risk factors: (i) a statistically significant positive association; (ii) a statistically significant inverse association; (iii) no statistically significant association.

## 2. DAL and Blood Pressure/Hypertension

Based on the results of numerous studies, it was found that diet-induced acidosis might contribute to elevated blood pressure. Table 1 shows the characteristics of studies referring to the association between DAL, expressed mainly by NEAP and PRAL, and systolic blood pressure (SBP), diastolic blood pressure (DBP) values and the prevalence of hypertension. This potential association between the acid–base status and elevated blood pressure was proved in a study by Kiefte-de Jong et al. [6], in which a higher prevalence of hypertension was reported in the highest vs. lowest quintile of estimated NEAP among three cohorts of participants without type 2 diabetes mellitus (T2DM), cardiovascular disease and cancer at baseline: The Nurses’ Health Study (NHS) (*n* = 67,433), The Nurses’ Health Study II (NHS-II) (*n* = 84,310) and The Health Professionals Follow-Up Study (HPFS) (*n* = 35,743). The greatest difference in the results concerning the prevalence of hypertension was observed in NHS-II conducted among women—15.2% vs. 9.3% (the highest vs. lowest quintile of NEAP). Moreover, the association between NEAP and the risk of hypertension was higher among lean women with the body mass index (BMI) < 25 kg/m^2^ (*p* < 0.001)—the multivariable relative risk (RR) for the top decile of NEAP was 1.27 (95% CI: 1.00–1.62) [13]. Similar results were obtained in a study performed by Murakami et al. [14] in female Japanese dietetic students. It was also shown that higher DAL, expressed as PRAL, was associated with the higher values of SBP and DBP after adjustment for possible confounding factors. Likewise, The Furukawa Nutrition and Health Study (*n* = 2028) by Akter et al. [3] showed that the odds ratio (OR) of hypertension in subjects in the highest tertile of PRAL and NEAP was 31% and 40% higher, respectively, than in individuals in the lowest tertile. Similar results indicating a higher prevalence of hypertension or elevated values of SBP and DBP in the highest vs. lowest PRAL and NEAP groups were shown in numerous other observational studies [4,5,15,16,17,18,19]. There are also several publications revealing differences in the statistical significance depending on the analyzed parameter. One such study was based on The Rotterdam Study by Engberink et al. [20], where SBP was significantly higher in the highest vs. lowest tertile of PRAL, while no significant difference was demonstrated in the case of DBP. Moreover, several studies revealed no difference [21,22,23,24,25,26], and only one study reported an inverse association between the prevalence of hypertension and the NEAP value [27]. Furthermore, in recent years, four systematic reviews and meta-analyses of observational studies demonstrated a significant positive association between DAL and hypertension. Detailed results are described in Table 1 [28,29,30,31].

Such an association between acid–base status and elevated blood pressure may be explained by the fact that metabolic acidosis caused by the Western diet impairs the absorption of minerals, which are effective in reducing blood pressure [32]. High DAL may increase the urinary excretion of calcium and magnesium and is inversely associated with intracellular potassium concentration [28,32]. Potassium deficiency causes compensatory sodium increase in cells to maintain volume and tonicity, which results in the aggravation of hypertension [33]. Furthermore, systemic metabolic acidosis may elevate blood pressure by raising cortisol secretion and lowering citrate excretion [34,35]. Moreover, the possible underlying mechanism of the described dependency is a decline in renal function [36]. Renal function is an important indicator and regulates acid–base balance—high DAL leads to increased renal ammoniagenesis and excretion of acid. It is beneficial for short periods to regulate acid–base homeostasis, but in the long term, it causes a degradation in renal function and might elevate blood pressure [36,37].

## 3. DAL and Carbohydrate Metabolism

It is well established that diet-related acidosis may also contribute to carbohydrate metabolism disorders, including an increased risk of insulin resistance and T2DM. Some studies have shown that high DAL is associated with significantly higher homeostasis model assessment of insulin resistance (HOMA-IR) [1,5,38,39]. A cross-sectional study by Moghadam et al. [15] revealed that high PRAL and NEAP values in adults significantly increase the risk of developing insulin resistance. The authors suggested that several mechanisms might be responsible for such an increased risk, e.g., excessive cortisol production, increased excretion of magnesium and reduced citrate excretion with urine as a result of high consumption of acid-forming products. It is suggested that acid–base balance disruption resulting from inappropriate dietary habits is also an important factor in the development of T2DM. Haghighatdoost et al. [4] observed that glycated hemoglobin was significantly higher in individuals from the highest category of acid load, expressed as PRAL, compared to those in the lowest category. Moreover, a study by Rebholz et al. [16] showed that the prevalence of T2DM in the highest quintile of PRAL was significantly higher compared to the lowest quintile. The observation was confirmed by a meta-analysis and a systematic review including 33 observational studies, which showed that the prevalence of T2DM was significantly higher in the highest quintile of DAL compared to the lowest one [29]. Furthermore, Fagherazzi et al. [40] conducted a study in a cohort of 66,485 women. It revealed that high NEAP and PRAL values increased the risk of T2DM by over 50% (HR 1.56, 95% CI: 1.29–1.90; *p* < 0.0001 for PRAL; RR 1.57, 95% CI: 1.30–1.89; *p* < 0.0001 for NEAP) with the correlation being particularly intensified in women with BMI < 25 kg/m^2^. Similar results were also obtained by Kiefte-de Jong et al. [6], who demonstrated a significant positive correlation between DAL and the risk of T2DM in all three studied cohorts (NHS, NHS II, HPFS). This conforms with the results of a meta-analysis and a systematic review including seven observational studies that enrolled 319,542 women and men. All high indices of acid load (PRAL, NEAP, A:P) were associated with a higher risk of T2DM in both sexes [41]. However, two studies revealed a sex-related difference in the obtained results [23,42]. Akter et al. [42] demonstrated that the correlation between the risk of T2DM and DAL was significant only in men, while no such correlation was observed in the studied women. Kucharska et al. [23] observed that the correlation between the occurrence of T2DM and NEAP value was significant only in women, while no such correlation was found in men. Notably, not all studies confirmed a positive relationship between DAL and carbohydrate metabolism. Numerous studies revealed no statistically significant correlation between the markers of carbohydrate metabolism and DAL [1,4,5,14,17,18,22,23,24,25,26,28,29,42,43,44], which is also true regarding the correlation between DAL and HOMA-IR values [5,28,29], fasting blood sugar [1,4,5,14,17,23,28,29,43,44] and glycated hemoglobin (HbA1c) [1,14,28,29]. Moreover, two papers did not confirm a relationship between DAL and the occurrence of insulin resistance [18,26], and six papers demonstrated no relationship with the occurrence of T2DM [22,23,24,25,26,42]. Furthermore, two cross-sectional studies revealed an inverse correlation between the markers of carbohydrate metabolism and DAL [4,27]. Detailed results concerning the correlation between DAL and carbohydrate metabolism are presented in Table 2.

## 4. DAL and Lipid Metabolism

According to numerous authors, lipid metabolism is also associated with DAL. Murakami et al. [14] demonstrated that the concentrations of total cholesterol and low-density lipoprotein (LDL-C) were significantly higher in all PRAL categories compared to the lowest ones (1925.0 ± 21.0 mg/L vs. 1866.0 ± 21 mg/L; *p* < 0.05 for total cholesterol, 1103.0 ± 18.0 mg/L vs. 1043.0 ± 18 mg/L; *p* < 0.05 for LDL-C). Other authors obtained similar results [5,21]. They also observed a significant relationship between the concentrations of LDL-C, total cholesterol and DAL. A similar correlation was demonstrated in a study by Haghighatdoost et al. [4] with reference to the concentration of triacylglycerol (TAG). Conversely, only two cross-sectional studies showed the correlation to be negative [4,45]. Haghighatdoost et al. [4] observed that LDL-C was significantly lower in the highest A:P category, while Krupp et al. [45] noted a similar correlation regarding the concentration of total cholesterol. However, the strongest correlation between lipid metabolism disorders and DAL was observed in the case of triglycerides (TG). Numerous authors demonstrated significantly higher TG values in the highest DAL categories (expressed as PRAL, NEAP, A:P) [5,17,21,23,46]. Moreover, Kucharska et al. [23] observed that the prevalence of hypertriglyceridemia was significantly higher in the highest NEAP category in women, while no such correlation was reported in men. Furthermore, a meta-analysis and a systematic review including 29 studies showed that high PRAL values were associated with TG concentrations that were higher by 3.47 mg/dL [46]. The authors suggested that it might be associated with excessive cortisol and insulin secretion as a result of consuming a diet characterized by high acid-forming potential. An inverse, statistically significant correlation was observed for high-density lipoprotein (HDL-C), which indicated that its significantly higher concentrations were related to the lowest DAL [5,17,23]. However, a pronounced majority of studies demonstrated the lack of correlation between DAL and the markers of lipid metabolism, including TG [4,5,8,14,15,21,22,23,24,25,26,28,44,46]. The observations were corroborated by the results of two meta-analyses and systematic reviews of 62 studies that showed no correlation between the markers of lipid metabolism and DAL. Moreover, a study by Xu et al. [26] and Kucharska et al. [23] showed no correlation between PRAL and the prevalence of hyperlipidemia in the studied participants. Detailed results concerning the correlation between DAL and lipid metabolism are presented in Table 3.

## 5. Discussion

The results of the analyzed studies are equivocal or even conflicting. This discrepancy is mainly regarding the influence of DAL on lipid and carbohydrate metabolism, whereas the correlation between DAL and blood pressure seems to be the most visible. The experimental confounders may contribute to these discrepancies in the results.

### 5.1. Demographic, Health and Lifestyle Confounders

Numerous authors presented data referring to patients constituting a homogeneous group regarding their sex, age or level of education [6,13,14,19,40,44]. However, such factors may influence the obtained results, and the homogeneity of the study group may limit the possibility of generalizing the results to the remaining population. A prospective cohort study by Akter et al. [42] showed that the correlation between the risk of developing T2DM and DAL was significant only in men, while no such correlation was observed in the studied women. Additionally, it was observed that the correlation was more distinct in the case of younger study participants. The relationship between the results and age might also be observed in a study by Luis et al. [24] and Xu et al. [26], in which the study groups included individuals aged 70–71. No correlation presented in these studies was statistically significant with regard to both the relationship between DAL and hypertension and between carbohydrate and lipid metabolism. Similar observations were presented in cross-sectional studies including individuals aged over 65 [22] and over 55 years [8]. Interestingly, the cited studies fully illustrated the analyzed data about the elderly, which may suggest that age has a significant influence on the obtained results. Another example of a study group’s homogeneity that may have influenced the obtained results is a diagnosed medical condition being the study inclusion criterion. The analyzed papers included several studies conducted in groups of patients with T2DM, diabetic nephropathy and chronic kidney disease at various stages [4,18,21,25,43]. It was demonstrated that the coexistence of such conditions as T2DM or other disorders of carbohydrate metabolism constituted a factor promoting the increase of DAL [47]. Moreover, patients with T2DM are commonly characterized by low physical activity and inappropriate dietary habits, which are not assessed with the PRAL or NEAP but may also affect metabolic control or lipid metabolism [47]. Furthermore, the participants of numerous cohort studies [6,13,40] were usually monitored for several years in order to confirm whether a medical condition developed over time. Notably, patients may have changed their lifestyle and nutrition during the observation period, which might have both improved and weakened the obtained results. Additionally, self-reporting the occurrence of the medical conditions by participants of some studies [3,40] might have contributed to the underestimation of the actual distribution in the study population.

### 5.2. Dietary Pattern/Nutritional Confounders

It is worth noting a considerable similarity between the low-DAL diet and Dietary Approaches to Stop Hypertension (DASH) which is recognized in the prophylaxis and treatment of hypertension [45,48]. The similarity might largely explain the consistency in the correlations between low DAL and a lower prevalence of hypertension. DASH is characterized by high potassium consumption and reduced DAL due to a high percentage of fruit and vegetable and a low percentage of meat product intake [45]. Some authors have suggested that the observed positive effects on health were mostly due to the implementation of diets based to a large extent on vegetables and that low DAL additionally enhanced the health benefits or might be treated as a marker of healthy nutrition [47,49].

On the other hand, it should be highlighted that there are some groups of products that are acidogenic according to the PRAL value, but for which several other mechanisms may make their activity beneficial in terms of metabolic control and lipid metabolism disorders [1]. This may disrupt the relationship between DAL and the risk of those disorders. The consumption of fish (PRAL ≈ 8 mEq/100 g) [1], particularly fatty sea fish, may illustrate this trend. These are a source of *n*-3 long-chain polyunsaturated fatty acids (PUFA), which have anti-inflammatory properties mainly related to reduced production of proinflammatory mediators from n-6 PUFA due to the competition for enzymes metabolizing those fatty acids. Another important factor is related to the diminished infiltration of the adipose tissue by macrophages and the change of their phenotype into an anti-inflammatory one (M2 polarized stage), which results in the decreased production of proinflammatory cytokines and increased release of anti-inflammatory ones [50]. Moreover, *n*-3 PUFAs are the substrate for the synthesis of anti-inflammatory resolvins, and they intensify the secretion of adiponectin by the adipose tissue [51]. All of these mechanisms may explain the beneficial influence of *n*-3 PUFA on insulin resistance and lipid metabolism disorders, particularly the serum concentration of TG [52].

Cereal products are characterized by similar PRAL values (4–7 mEq/100 g), regardless of the type of flour they are made from, which is associated with dietary fiber content [1]. However, an extensive review of prospective studies showed that high consumption of cereal fiber was linked to the reduced risk of not only T2DM, but also obesity and cardiovascular diseases [53]. This may be explained by the beneficial properties of short-chain fatty acids (SCFA) produced in the intestinal bacterial fermentation of dietary fiber on the intestinal microbiota, which exerts a positive effect on glucose tolerance and alleviates systemic inflammation [54]. Furthermore, a significant role is ascribed to insoluble cereal fiber. Its consumption is connected with the hindered absorption of dietary protein that presents insulinotropic properties, which results in the reduced resistance of tissues to insulin and the risk of developing T2DM [55].

Dairy products are generally considered as acidogenic with high PRAL values, as they are the source of numerous protein components [1]. Nevertheless, some authors have suggested that the consumption of milk and milk products, particularly yogurt, may prove beneficial for the control of glycemia, insulin secretion, tissue sensitivity to insulin and reducing the risk of T2DM [56,57,58]. This can be explained by the fact that dairy products also contain bioactive peptides, vitamins, minerals and carbohydrates with low glycemic index, which all appear to have a favorable effect on the control of glycemia [59]. Moreover, the consumption of yogurt and other fermented milk products exerts a positive influence on the intestinal microbiota and therefore on tissues’ insulin sensitivity [60]. Enhancing dairy products with probiotics and vitamin D may additionally strengthen this activity [61,62]. It is also worth noting that milk products may stimulate insulin secretion, due to their high content of whey proteins rich in branched-chain amino acids. This may lead to insulin resistance in the long term, but it may temporarily decrease postprandial glycemia [63]. Therefore, it may be concluded that hyperinsulinemia caused by the consumption of milk products in patients with disrupted carbohydrate metabolism may even be beneficial in the control of glycemia [58].

There are also some products, such as sweets, sweet beverages or confectioneries, which have a relatively low PRAL value but at the same time have a high glycemic index and are a source of unsaturated trans fatty acids. As a result, these products exert a negative effect on carbohydrate and lipid metabolism [64,65,66]. Similarly, alcohol is a product characterized by low PRAL (PRAL ≈ −2 mEq/100 g) [1], but according to the literature, its consumption is associated with increased serum TG and blood pressure [52]. However, it seems that moderate alcohol consumption may be beneficial in the prophylaxis of T2DM and cardiovascular diseases, which is probably due to increased serum HDL-C [52,67] and reduction in fasting glucose levels and HbA_1c_, and increased insulin sensitivity [68].

All the described mechanisms of action regarding the above-mentioned alimentary products, and possibly numerous other products, may impede the assessment of the correlation between DAL and the studied metabolic disorders. It may be speculated that this may underlie the discrepancies regarding the results presented in this paper.

## 6. Conclusions

It seems that high DAL negatively affects cardiometabolic risk factors. This has particularly been confirmed in case of blood pressure—elevated SBP and DBP—and the prevalence of hypertension. It may be related to the fact that a low-DAL diet is partially similar to DASH, which is well recognized in the treatment of hypertension. The association between DAL and carbohydrate metabolism seems to be weaker, but it was still confirmed in numerous studies including the meta-analyses of observational studies. However, due to the lack of unambiguous evidence and multiple experimental confounders, such as nutritional, demographic and health confounders, more studies are necessary to verify the potential relationship between DAL and lipid profile.

## Figures and Tables

**Table 1:** Characteristics of studies referring to the association between DAL, systolic blood pressure (SBP), diastolic blood pressure (DBP) and the prevalence of hypertension.

First Author/Country/Reference Number	Year	Study Design	Sample (*n*)	Gender/Age Range/Mean Age	DAL Assessment Method Related to the Result	Dietary Intake Assessment Tool	Result
**Statistically significant positive association**							
Murakami et al./Japan [14]	2008	Cross-sectional	1136 (dietetic students)	Women, 18–22 y	PRAL, A:P	BDHQ	Higher PRAL and A:P associated with higher SBP and DBP values (*p* for trend = 0.028 and 0.035 for PRAL, and 0.012 and 0.009 for A:P, respectively).
Zhang et al./the USA [13]	2009	Cohort—NHS-II	87 293 (women without prior hypertension)	Women, 31–41 y	NEAP, A:P	FFQ	NEAP and A:P positively associated with the risk of hypertension (RR: 1.14, 95% CI: 1.05–1.24 for NEAP and RR: 1.23, 95% CI: 1.08–1.41 for A:P, respectively). The association between NEAP and risk of hypertension higher among lean women with BMI < 25 kg/m^2^ (*p* < 0.001).
Engberink et al./ the Netherlands [20] *	2012	Cross-sectional baseline data	2241 (participants without hypertension at baseline)	Both, ≥55 y	PRAL	FFQ	SBP significantly higher in the highest vs. lowest tertile of PRAL (122.4 ± 11.7 mmHg vs. 121.1 ± 12.2 mmHg; *p* < 0.05).
Krupp et al./Germany [19]	2013	Cross-sectional	267 (children)	Both, 4–14 y	PRAL, NAE	3d-FD	SBP increased by 4.3 mmHg and 3.2 mmHg from the lowest to the highest tertiles of PRAL and NAE, respectively. DAL significantly (*p* < 0.01) directly related to SBP.
Akter et al./Japan [3]	2015	Cross-sectional	2028 (working population)	Both, 18–70 y	PRAL, NEAP	BDHQ	The increased risk of hypertension in the highest vs. lowest tertile of PRAL (OR: 1.31; Cl: 1.01–1.70) and NEAP (OR: 1.40; CI: 1.08–1.82).
Haghighatdoost et al./Iran [4]	2015	Cross-sectional	547 (patients with diabetic nephropathy)	Both, 66.8 y (mean age)	PRAL	FFQ	SBP significantly higher in the highest vs. lowest PRAL categories (106.1 ± 0.7 mmHg vs. 103.6 ± 0.7 mmHg; *p* < 0.05).
Rebholz et al./the USA [16]	2015	Cross-sectional	15,055 (general population)	Both, 54 y (mean age)	PRAL	FFQ	The prevalence of hypertension significantly higher in the highest vs. lowest PRAL quartile (37.7% vs. 31.9%).
Bahadoran et al./Iran [17] *	2015	Cross-sectional	5620 (general population)	Both, 19–70 y	PRAL, A:P	147-item FFQ	DBP positively associated with dietary PRAL (standardized *β* coefficient = 0.062; *p* < 0.01) and A:P ratio (standardized *β* coefficient = 0.026; *p* < 0.05).
Han et al./Korea [5]	2016	Cross-sectional	11,601 (subjects without prior CVD)	Both, 40–79 y	PRAL, NEAP	24HR	PRAL and NEAP positively associated with the prevalence of hypertension (OR: 1.19, 95% CI: 1.09–1.31), as well as SBP and DBP values.
Moghadam et al./Iran [15] *	2016	Cross-sectional	925 (general population)	Both, 22–80 y	PRAL	FFQ	DBP significantly higher in the highest vs. lowest quartile of PRAL (73.5 ± 10.5 mmHg vs. 72.8 ± 10.9 mmHg; *p* < 0.05).
Ikizler et al./the USA [18]	2016	Cross-sectional	42 (patients with chronic kidney disease stages 3–5)	Both, 60.8 (mean age)	NEAP	3-day prospective FD	SBP and DBP significantly higher in the highest vs. lowest NEAP tertile (133 mmHg vs. 131 mmHg and 80.1 mmHg vs. 78.5 mmHg, respectively).
Kiefte-de Jong et al./the USA [6] *	2017	Cohort—NHS Follow-up data	67,433 (women without T2DM, CVD and cancer at baseline)	Women, 30–55 y	NEAP	FFQ	A higher prevalence of hypertension in the highest vs. lowest categories of NEAP (24.4% vs. 21.3%).
		Cohort—NHS-II Follow-up data	84,310 (women without T2DM, CVD and cancer at baseline)	Women, 25–42 y			A higher prevalence of hypertension in the highest vs. lowest categories of NEAP (15.2% vs. 9.3%).
		Cohort—HPFS Follow-up data	35,743 (men without T2DM, CVD and cancer at baseline)	Men, 40–75 y			A higher prevalence of hypertension in the highest vs. lowest categories of NEAP (36.6% vs. 35.6%).
Daneshzad et al./Iran [28]	2019	A systematic review and meta-analysis of observational studies (16 cohort studies; 17 cross-sectional studies)	92,478	Both, >1 y	NEAP, PRAL, NAE	All mentioned assessment tools	A significant relationship between SBP (WMD = 1.74, 95% CI: 0.25–3.24 mmHg; *p* < 0.05), DBP (WMD = 0.75, 95% CI: 0.07–1.42 mmHg; *p* < 0.05) and DAL in cross-sectional studies.
Parohan et al./Iran [31] *	2019	A systematic review and meta-analysis of observational studies (3 cohort studies; 11 cross-sectional studies)	306,183	Both, >18 y	PRAL	All mentioned assessment tools	A significant association between PRAL and hypertension in prospective studies (*p* < 0.01) In linear dose-response analysis, a 20-unit increase in PRAL values associated with the risk of hypertension increased by 3% (combined effect size: 1.03, 95% CI: 1.00–1.06, *p* < 0.05).
Shao-Wei et al./China [30]	2019	A systematic review and meta-analysis of observational studies (4 cohort studies; 6 cross-sectional studies)	135,072	Both, >18 y	PRAL, NEAP	All mentioned assessment tools	Hypertension significantly associated with higher PRAL (OR = 1.14, 95% CI: 1.02–1.17). PRAL categories associated with higher DBP (WMD = 0.96, 95% CI: 0.67–1.26) and SBP (WMD = 1.57, 95% CI: 1.12–2.03). A 35% increase in the risk of hypertension associated with higher NEAP (OR = 1.35, 95% CI: 1.03–1.78).
Dehghan et al./Iran [29]	2020	A systematic review and meta-analysis of observational studies (2 cohort studies; 12 cross-sectional studies)	519,262	Both, >18 y	PRAL	All mentioned assessment tools	The highest PRAL categories associated with higher SBP (WMD = 0.98, 95% CI: 0.51, 1.45 mmHg; *p* < 0.001) and DBP (WMD = 0.61, 95% CI: 0.09, 1.14 mmHg; *p* < 0.05).
**Statistically significant inverse association**							
Amodu et al./the USA [27]	2013	Cross-sectional	13,274 (general population)	Both, ≥20 y	NEAP	24HR	The prevalence of hypertension in the lowest categories of NEAP significantly higher than in the highest one (43.5% vs. 34.9%, *p* < 0.001).
**No statistically significant association**							
van-den Berg et al./Denmark [25]	2012	Cross-sectional	707 (renal transplant patients)	Both, 53 y (mean age)	NEAP	FFQ	No difference the prevalence of hypertension, SBP, DBP and between the tertiles of NEAP.
Engberink et al./the Netherlands [20] *	2012	Cross-sectional baseline data	2241 (participants without hypertension at baseline)	Both, ≥55 y	PRAL	FFQ	No significant difference in DBP in the highest vs. lowest PRAL tertile.
Luis et al./Sweden [24]	2014	Cross-sectional	673 (general population)	Male, 70–71 y	PRAL	7d-FD	No significant difference in the prevalence of hypertension, SBP, DBP, and between the tertiles of PRAL. No significant difference in SBP, DBP after 7 y follow-up and cross-sectionally between the quartiles of PRAL.
Iwase et al./Japan [21]	2015	Cross-sectional	149 (patients with T2DM)	Both, 65.7 ± 9.3 (mean age)	PRAL, NEAP	BDHQ	No significant difference in SBP in the highest vs. lowest PRAL and NEAP tertile.
Bahadoran et al./Iran [17] *	2015	Cross-sectional	5620 (general population)	Both, 19–70 y	PRAL, A:P	147-item FFQ	No significant association between SBP and dietary PRAL, as well as A:P ratio.
Moghadam et al./Iran [15] *	2016	Cross-sectional	925 (general population)	Both, 22–80 y	PRAL	FFQ	No significant difference in SBP, DBP after 3 y follow-up between PRAL categories.
Xu et al./Sweden [26]	2016	Cross-sectional	911 (general population)	Both, 70–71 y	PRAL	7d-FD	No significant difference in the prevalence of hypertension between PRAL categories.
Ko et al./Korea [22]	2017	Cross-sectional	1369 (general population)	Both, ≥65 y	NEAP	FFQ	No significant difference in the prevalence of hypertension, SBP, DBP and between the lowest and highest NEAP categories.
Kucharska et al./Poland [23]	2018	Cross-sectional	6170 (general population)	Both, ≥20 y	NEAP, PRAL	24HR	No significant differences in the prevalence of hypertension and SBP, DBP across PRAL and NEAP tertiles.
Parohan et al./Iran [31] *	2019	A systematic review and meta-analysis of observational studies (3 cohort studies; 11 cross-sectional studies)	306,183	Both, >18 y	PRAL	All mentioned assessment tools	No significant association between PRAL and hypertension in cross-sectional studies.

Abbreviations: y: year; 24HR, 24-h dietary recall questionnaire; A:P, animal-protein-to-potassium ratio; BDHQ, brief validated self-administered diet history questionnaire; DBP, diastolic blood pressure; HR, hazard ratio; NEAP, net-endogenous acid production; PRAL, potential renal acid load; SBP, systolic blood pressure; FD, food diary; FFQ, food frequency questionnaire; NAE, urine net acid excretion; T2DM, type 2 diabetes mellitus; DAL, dietary acid load; WMD, weighted mean difference; CVD, cardiovascular disease. * Indicates consecutive outcomes that stemmed from one study, but differences in the significance of association between blood pressure values and DAL were obtained.

**Table 2:** Characteristics of studies referring to the association between DAL, fasting blood sugar, glycated hemoglobin (HbA1c), type 2 diabetes mellitus (T2DM), insulin resistance, insulin sensitivity, homeostasis model assessment of insulin resistance (HOMA-IR) and fasting insulin.

First Author/Country/Reference Number	Year	Study Design	Sample (*n*)	Gender/Age Range/Mean Age	DAL Assessment Method Related to the Result	Dietary Intake Assessment Tool	Result
**Statistically significant positive association**							
Fagherazzi et al./France [40]	2014	Cohort study	66,485 (general population)	Women, >18y	PRAL, NEAP	208-item diet-history questionnaire	NEAP and PRAL positively associated with the risk of T2DM (HR 1.56, 95% CI: 1.29–1.90; *p* < 0.0001 for PRAL, RR 1.57, 95% CI: 1.30–1.89; *p* < 0.0001 for NEAP). PRAL and NEAP: a stronger association in women with BMI < 25 kg/m^2^ (HR 1.96, 95% CI: 1.43–2.69; *p* < 0.001 for PRAL, HR 2.04, 95% CI: 1.50–2.76; *p* < 0.0001 for NEAP) than in overweight women (HR 1.28, 95% CI: 1.00–1.64; *p* = 0.05 for PRAL, HR 1.25; 95% CI: 0.98–1.60; *p* = 0.05 for NEAP).
Haghighatdoost et al./Iran [4] *	2015	Cross-sectional	547 (patients with diabetic nephropathy)	Both, 66.8 y (mean age)	PRAL, A:P	FFQ	HbA_1c_ significantly higher in the highest vs. lowest PRAL categories (7.8 ± 0.5% vs. 5.7 ± 0.5%; *p* = 0.01). HbA_1c_ significantly higher in the highest vs. lowest A:P categories (7.6 ± 0.5% vs. 5.8 ± 0.5%; *p* = 0.03).
Rebholz et al./the USA [16]	2015	Cross-sectional	15,055 (general population)	Both, 54 y (mean age)	PRAL	FFQ	A significantly higher prevalence of T2DM in the highest vs. lowest PRAL quartile (13.8% vs. 10%).
Akter et al./Japan [3] *	2016	Cross-sectional	1732 (working population)	Both, 19–69 y	PRAL, NEAP	BDHQ	PRAL and NEAP positively associated with HOMA-IR. Positive associations limited to subjects with BMI < 23 kg/m^2^.
Han et al./Korea [5] *	2016	Cross-sectional	11,601 (general population)	Both, 40–79 y	PRAL, NEAP	24HR	Insulin significantly higher in the highest vs. lowest NEAP categories (10.4 ± 6.7 μU/mL vs. 9.5 ± 4.8 μU/mL; *p* < 0.001). HOMA-IR significantly higher in the highest vs. lowest NEAP tertile (2.6 ± 2.6 vs. 2.4 ± 1.8; *p* < 0.001).
Kiefte-de Jong et al./the USA [6]	2016	Cohort—NHS Follow-up data	67,433 (women without T2DM, CVD and cancer at baseline)	Women, 30–55 y	PRAL, NEAP, A:P	FFQ	NEAP, PRAL and A:P positively associated with the risk of T2DM (HR 1.28, 95% CI: 1.18–1.38; *p* < 0.0001 for NEAP, RR 1.26, 95% CI: 1.16–1.36; *p* < 0.0001 for PRAL and HR 1.26, 95% CI: 1.16–1.36; *p* < 0.0001 for A:P.
		Cohort—NHS-II Follow-up data	84,310 (women without T2DM, CVD and cancer at baseline)	Women, 25–42 y			PRAL, NEAP and A:P positively associated with the risk of T2DM (HR 1.30, 95% CI: 1.17–1.44; *p* < 0.0001 for NEAP, RR 1.33, 95% CI: 1.20–1.48; *p* < 0.0001 for PRAL and HR 1.35, 95% CI: 1.21–1.50; *p* < 0.0001 for A:P.
		Cohort—HPFS Follow-up data	35,743 (men without T2DM, CVD and cancer at baseline)	Men, 40–75 y			PRAL, NEAP and A:P positively associated with the risk of T2DM (HR 1.32, 95% CI: 1.18–1.47; *p* < 0.0001 for NEAP, HR 1.29, 95% CI: 1.16–1.44; *p* < 0.0001 for PRAL and HR 1.39, 95% CI: 1.25–1.55; *p* < 0.0001 for A:P.
Moghadam et al./Iran [15] *	2016	Cross-sectional	925 (general population)	Both, 22–80 y	PRAL, NEAP	FFQ	PRAL and NEAP positively associated with the risk of insulin resistance (OR 2.81, 95% CI: 1.32–5.97; *p* = 0.005 for PRAL an OR 2.18, 95% CI: 1.03–4.61; *p* = 0.021 for NEAP).
Akter et al./Japan [42] *	2016	Cross-sectional	27,809 (general population)	Men, 56.5 y (mean age)	PRAL, NEAP	147-item FFQ	PRAL positively associated with the risk of T2DM (OR 1.61, 95% CI: 1.16–1.24; *p* < 0.01 for PRAL.
			36,851 (general population)	Women, 53.8 y (mean age)			
Gæde et al./Denmark [39]	2018	Cross-sectional	56,479 (general population)	Both, 30–64 y	PRAL	FFQ	PRAL positively associated with the risk of T2DM (HR 1.24, 95% CI: 1.14–1.35; *p* < 0.05). HOMA-IR, PG at 120 min, fasting insulin and insulin at 120 min significantly higher in the highest vs. lowest PRAL categories.
Kucharska et al./Poland [23] *	2018	Cross-sectional	2760 (general population)	Men,49 y (mean age)	NEAP, PRAL	24HR	
			3409 (general population)	Women, 52 y (mean age)			A significantly higher prevalence of T2DM in the highest vs. lowest NEAP quartile (11.33% vs. 8.47%; *p* for trend = 0.05). Fasting blood sugar significantly higher in the highest vs. lowest NEAP categories (5.4 vs. 5.07 mmol/L; *p* < 0.01).
Banerjee et al./the USA [38]	2018	Cross-sectional	3257 (African-Americans from the Jackson)	Both, 21–84 y	PRAL, NAE	FFQ	HOMA-IR significantly higher in the highest vs. lowest PRAL categories (90.9 ± 8.7 vs. 75.8 ± 8.6; *p* = 0.03).
Jayedi et al./Iran [41]	2018	A systematic review and dose-response meta-analysis of prospective observational studies (7 cohort studies)	319,542 (general population)	Both, >18 y	PRAL, NEAP, A:P	All mentioned assessment tools	NEAP, PRAL and A:P positively associated with the risk of T2DM (HR 1.03, 95% CI: 1.01–1.04; *p* = 0.04 for NEAP, HR 1.04, 95% CI: 1.01–1.06; *p* < 0.0001 for PRAL and HR 1.11, 95% CI: 1.07–1.15; *p* < 0.05 for A:P).
Dehghan et al./Iran [29] *	2020	A systematic review and meta-analysis of observational studies (2 cohort studies; 12 cross-sectional studies)	519,262 (general population)	Both, >18 y	PRAL	All mentioned assessment tools	The highest PRAL categories associated with higher insulin (WMD = −0.235, 95% CI: 0.070–0.400 μIU/mL; *p* < 0.005), higher odds of T2DM (OR 1.19, 95% CI: 1.092–1.311; *p* < 0.001) and a higher prevalence of T2DM (13% and 11% in the highest vs. lowest category).
**Statistically significant inverse association**							
Amodu et al./the USA [27]	2013	Cross-sectional	13,274 (general population)	Both, ≥20 y	NEAP	24HR	The prevalence of T2DM in the lowest quartile of NEAP significantly higher than in the highest (7.7% vs. 6.2%; *p* < 0.05).
Haghighatdoost et al./Iran [4] *	2015	Cross-sectional	547 (patients with diabetic nephropathy)	Both, 66.8 y (mean age)	PRAL, A:P	FFQ	Fasting blood sugar significantly lower in the highest vs. lowest PRAL categories (129.4 ± 1.0 mg/dL vs. 133.7 ± 1.0 mg/dL; *p* = 0.01).
**No statistically significant association**							
Murakami et al./Japan [14]	2008	Cross-sectional	1136 (dietetic students)	Women, 18–22 y	PRAL, A:P	BDHQ	No significant association between fasting blood sugar, HbA_c1_ and dietary PRAL, as well as A:P ratio.
van-den Berg et al./Denmark [25]	2012	Cross-sectional	707 (renal transplant patients)	Both, 53 y (mean age)	NAE	FFQ	No significant difference in the prevalence of T2DM across the tertiles of NAE.
Luis et al./Sweden [24]	2014	Cross-sectional	673 (general population)	Men, 70–71 y	PRAL	7d-FD	No significant difference in the prevalence of T2DM across the tertiles of PRAL.
Bahadoran et al./Iran [17]	2015	Cross-sectional	5620 (general population)	Both, 19–70 y	PRAL, A:P	147-item FFQ	No significant association between fasting blood sugar and dietary PRAL, as well as A:P ratio.
Haghighatdoost et al./Iran [4] *	2015	Cross-sectional	547 (patients with diabetic nephropathy)	Both, 66.8 y (mean age)	PRAL, A:P	FFQ	No significant association between fasting blood sugar and A:P ratio.
Han et al./Korea [5] *	2016	Cross-sectional	11,601 (general population)	Both, 40–79 y	PRAL, NEAP	24HR	No significant difference in HOMA-IR, fasting blood sugar and insulin in the highest vs. lowest PRAL tertile. No significant difference in fasting blood sugar in the highest vs. lowest NEAP tertile.
Xu et al./Sweden [26]	2016	Cross-sectional	911 (general population)	Both, 70–71 y	PRAL	7d-FD	No significant difference in the prevalence of T2DM and insulin sensitivity between PRAL tertiles.
Ikizler et al./the USA [18]	2016	Cross-sectional	42 (patients with chronic kidney disease stages 3–5)	Both, 60.8 (mean age)	NEAP, PRAL	3-day prospective FD	No significant association between insulin sensitivity and dietary PRAL, as well as NEAP.
Akter et al./Japan [1] *	2016	Cross-sectional	1732 (working population)	Both, 19–69 y	PRAL, NEAP	BDHQ	No significant association between DAL score and fasting blood sugar or HbA_1c_ levels.
Akter et al./Japan [42] *	2016	Cross-sectional	27,809 (general population)	Men, 56.5 y (mean age)	PRAL, NEAP	147-item FFQ	No significant association between T2DM and dietary NEAP.
			36,851 (general population)	Women, 53.8 y (mean age)			No significant difference in the prevalence of T2DM and dietary NEAP and PRAL.
Ko et al./Korea [22]	2017	Cross-sectional	1369 (general population)	Both, ≥65 y	NEAP	FFQ	No significant difference in the prevalence of T2DM across the quartiles of NEAP.
Kucharska et al./Poland * [23]	2018	Cross-sectional	2760 (general population)	Men, 49 y (mean age)	NEAP, PRAL	24HR	No significant differences in the prevalence of T2DM across the tertiles of PRAL and NEAP. No significant association between fasting blood sugar and dietary PRAL, as well as NEAP.
			3409 (general population)	Women, 52 y (mean age)			No significant differences across the tertiles of PRAL concerning the prevalence of T2DM. No significant association between fasting blood sugar and dietary PRAL.
Daneshzad et al./Iran [28]	2019	A systematic review and meta-analysis of observational studies (16 cohort studies; 17 cross-sectional studies)	92,478 (general population)	Both, >1 y	NEAP, PRAL, NAE	All mentioned assessment tools	No significant association between fasting blood sugar, HbA_1c_, serum insulin, HOMA-IR and dietary PRAL, NAE, as well as NEAP.
Kabasawa et al./Japan [43]	2019	Cross-sectional	6684 (patients with chronic kidney disease)	Both, 40–97 y	PRAL, NEAP	FFQ	No significant association between fasting blood sugar and dietary PRAL, as well as NEAP.
Mozaffari et al./Iran [44]	2019	Cross-sectional	371 (Iranian healthy women	Women, 20–50 y	NEAP, PRAL	FFQ	No significant association between fasting blood sugar and dietary PRAL, as well as NEAP.
Dehghan et al./Iran [29] *	2020	A systematic review and meta-analysis of observational studies (2 cohort studies; 12 cross-sectional studies)	519,262 (general population)	Both, >18 y	PRAL	All mentioned assessment tools	No significant association between fasting blood sugar, HbA_1c_, HOMA-IR and dietary PRAL, as well as NEAP.

**Abbreviations**: 24HR, 24-h dietary recall questionnaire; A:P, animal-protein-to-potassium ratio; BDHQ, brief validated self-administered diet history questionnaire; HbA_1c_, glycated hemoglobin; HOMA-IR, homeostasis model assessment of insulin resistance; HR, hazard ratio; NEAP, net-endogenous acid production; PRAL, potential renal acid load; FD, food diary; FFQ, food frequency questionnaire; NAE, urine net acid excretion; BMI, body mass index; T2DM, type 2 diabetes mellitus; DAL, dietary acid load; WMD, weighted mean difference; CVD, cardiovascular disease; PG, plasma glucose. * Indicates consecutive outcomes that stemmed from one study but differences in the significance of association between carbohydrate metabolism and DAL were obtained.

**Table 3:** Characteristics of studies referring to the association between DAL, triacylglycerol (TAG), low-density lipoprotein (LDL-C), high-density lipoprotein (HDL-C), total cholesterol, triglyceride (TG)**.**

First Author/Country/Reference Number	Year	Study Design	Sample (*n*)	Gender/Age Range/Mean Age	DAL Assessment Method Related to the Result	Dietary Intake Assessment Tool	Result
**Statistically significant positive association**							
Murakami et al./Japan [14]	2008	Cross-sectional	1136 (dietetic students)	Women, 18–22 y	PRAL, A:P	BDHQ	Total cholesterol, LDL-C significantly higher in the highest vs. lowest PRAL categories (1925.0 ± 21.0 mg/L vs. 1866.0 ± 21 mg/L; *p* = 0.042 for total cholesterol, 1103.0 ± 18.0 mg/L vs. 1043.0 ± 18 mg/L; *p* = 0.021 for LDL-C).
Haghighatdoost et al./Iran [4] *	2015	Cross-sectional	547 (patients with diabetic nephropathy)	Both, 66.8 y (mean age)	PRAL, A:P	FFQ	TAG significantly higher in the highest vs. lowest PRAL categories (257.4 ± 2.3 mg/dL vs. 146.9 ± 2.3 mg/dL; *p* = 0.006).
Bahadoran et al./Iran [17] *	2015	Cross-sectional	5620 (general population)	Both, 19–70 y	PRAL, A:P	147-item FFQ	PRAL and A:P positively associated with TG (*β* = 0.143, *p* < 0.01 for PRAL, *β* = 0.03, *p* < 0.05 for A:P).
Iwase et al./Japan [21] *	2015	Cross-sectional	149 (patients with T2DM)	Both, 65.7 ± 9.3 (mean age)	PRAL, NEAP	BDHQ	LDL-C, TG higher in the highest vs. lowest PRAL tertile (2.7 ± 0.8 mmol/L vs. 2.5 ± 0.8 mmol/L; *p* = 0.05 for LDL-C, 1.7 ± 1.1 mmol/L vs. 1.3 ± 0.7 mmol/L; *p* = 0.03 for TG). TG higher in the highest vs. lowest NEAP tertile (1.7 ± 1.2 mmol/L vs. 1.3 ± 0.7 mmol/L; *p* = 0.005).
Han et al./Korea [5] *	2016	Cross-sectional	11,601 (general population)	Both, 40–79 y	PRAL, NEAP	24HR	TG higher in the highest vs. lowest PRAL tertile (144.7 ± 113.5 mg/dL vs. 138.8 ± 102.7 mg/dL; *p* = 0.004). LDL-C higher in the highest vs. lowest PRAL tertile (119.0 ± 32.3 mg/dL vs. 119.0 ± 32.4 mg/dL; *p* = 0.043). TG higher in the highest vs. lowest NEAP tertile (148.7 ± 118.9 mg/dL vs. 137.4 ± 110.9 mg/dL; *p* < 0.001).
Kucharska et al./Poland * [23]	2018	Cross-sectional	2760 (general population)	Men, 49 y (mean age)	NEAP, PRAL	24HR	
			3409 (general population	Women, 52 y (mean age)			The prevalence of hypertriglyceridemia significantly higher in the highest vs. lowest quartile of NEAP (22.33% vs. 18.82%; *p* < 0.01). TG significantly higher in the highest vs. lowest NEAP categories (1.18 vs. 1.13 mmol/L; *p* < 0.05).
Farhangi et al./Iran [46] *	2019	A systematic review and meta-analysis (17 observational studies)	181,282 (general population)	Both, >18 y	PRAL, NEAP	All mentioned assessment tools	High PRAL associated with serum TG concentrations higher by 3.47 mg/dL (WMD: 3.468; CI: −0.231, 7.166, *p* < 0.05).
**Statistically significant inverse association**							
Haghighatdoost et al./Iran [4] *	2015	Cross-sectional	547 (patients with diabetic nephropathy)	Both, 66.8 y (mean age)	PRAL, A:P	FFQ	LDL-C significantly lower in the highest vs. lowest A:P categories (129.4 ± 1.6 mg/dL vs. 140.1 ± 1.6 mg/dL; *p* < 0.0001).
Bahadoran et al./Iran [17] *	2015	Cross-sectional	5620 (general population)	Both, 19–70 y	PRAL, A:P	147-item FFQ	PRAL and A:P inversely associated with HDL-C (*β* = –0.11, *p* < 0.01 for PRAL, *β* = −0.06, *p* < 0.01 for A:P).
Han et al./Korea [5] *	2016	Cross-sectional	11,601 (general population)	Both, 40–79 y	PRAL, NEAP	24HR	HDL-C significantly lower in the highest vs. lowest NEAP tertiles (50.7 ± 12.3 mg/dL vs. 51.5 ± 12.4 mg/dL, *p* = 0.031).
Kucharska et al./Poland [23] *	2018	Cross-sectional	2760 (general population)	Men,49 y (mean age)	PRAL, NEAP	24H	HDL-C significantly lower in the highest vs. lowest PRAL and NEAP categories (1.24 vs. 1.26 mmol/L; *p* < 0.01 for PRAL, 1.25 vs. 1.28 mmol/L; *p* < 0.05 for NEAP).
			3409 (general population	Women, 52 y (mean age)			HDL-C significantly lower in the highest vs. lowest PRAL and NEAP categories (1.53 vs. 1.50 mmol/L; *p* < 0.05 for PRAL, 1.51 vs. 1.54 mmol/L; *p* < 0.05 for NEAP).
Krupp et al./Germany [45]	2018	Cross-sectional	6797 (general population)	Both, >18 y	PRAL	FFQ	Total cholesterol significantly lower in the highest vs. lowest PRAL categories (192 mg/dL vs. 203.6 mg/dL; *p* < 0.0001).
**No statistically significant association**							
Murakami et al./Japan [14]	2008	Cross-sectional	1136 (dietetic students)	Women, 18–22 y	PRAL, A:P	BDHQ	No significant association between HDL-C, TAG and dietary PRAL. No significant association between total cholesterol, HDL-C, LDL-C, TAG and dietary A:P.
Engberink et al./the Netherlands [8]	2012	Cross-sectional baseline data	2241 (participants without hypertension at baseline)	Both, ≥55 y	PRAL	FFQ	No significant association between total cholesterol, HDL-C and dietary PRAL.
van-den Berg et al./Denmark [25]	2012	Cross-sectional	707 (renal transplant patients)	Both, 53 y (mean age)	NAE	FFQ	No significant difference between the tertiles of NAE, HDL-C and TG.
Luis et al./Sweden [24]	2014	Cross-sectional	673 (general population)	Men, 70–71 y	PRAL	7d-FD	No significant difference in the prevalence of hyperlipidemia between the tertiles of PRAL.
Haghighatdoost et al./Iran [4] *	2015	Cross-sectional	547 (patients with diabetic nephropathy)	Both, 66.8 y (mean age)	PRAL, A:P	FFQ	No significant association between total cholesterol, HDL-C and dietary PRAL. No significant association between TAG, total cholesterol and dietary A:P.
Iwase et al./Japan [21] *	2015	Cross-sectional	149 (patients with T2DM)	Both,65.7 ± 9.3 (mean age)	PRAL, NEAP	BDHQ	No significant association between total cholesterol, LDL-C and dietary PRAL.
Han et al./Korea [5] *	2016	Cross-sectional	11,601 (general population)	Both, 40–79 y	PRAL, NEAP	24HR	No significant association between LDL-C and dietary PRAL. No significant association between total cholesterol, LDL-C and dietary NEAP.
Moghadam et al./Iran [15] *	2016	Cross-sectional	925 (general population)	Both, 22–80 y	PRAL, NEAP	FFQ	No significant association between HDL-C, LDL-C, TG and dietary PRAL.
Xu et al./Sweden [26]	2016	Cross-sectional	911 (general population)	Both, 70–71 y	PRAL	7-d FD	No significant difference in the prevalence of hypercholesterolemia between PRAL tertiles.
Ko et al./Korea [22]	2017	Cross-sectional	1369 (general population)	Both, ≥65 y	NEAP	FFQ	No significant association between total cholesterol, TG and dietary NEAP.
Kucharska et al./Poland * [23]	2018	Cross-sectional	2760 (general population)	Men, 49 y (mean age)	NEAP, PRAL	24HR	No significant differences in the prevalence of hypercholesterolemia and hypertriglyceridemia across the tertiles of PRAL and NEAP. No significant association between total cholesterol, LDL-C, TG and dietary PRAL, as well as NEAP.
			3409 (general population)	Women, 52 y (mean age)			No significant differences in the prevalence of hypercholesterolemia and hypertriglyceridemia across the tertiles of PRAL. No significant differences across the tertiles of NEAP concerning the prevalence of hypercholesterolemia. No significant association between total cholesterol, LDL-C, TG and dietary PRAL. No significant association between total cholesterol, LDL-C and dietary NEAP.
Daneshzad et al./Iran [28]	2019	A systematic review and meta-analysis of observational studies (16 cohort studies; 17 cross-sectional studies)	92,478 (general population)	Both, >1 y	NEAP, PRAL, NAE	All mentioned assessment tools	No significant association between total cholesterol, HDL-C, LDL-C, TAG and dietary PRAL, NAE, as well as NEAP.
Mozaffari et al./Iran [44]	2019	Cross-sectional	371 (Iranian healthy women)	Women, 20–50 y	NEAP, PRAL	FFQ	No significant association between total cholesterol, LDL-C, HDL-C, TG and dietary PRAL, as well as NEAP.
Farhangi et al./Iran [46]	2019	A systematic review and meta-analysis (17 observational studies)	181,282 (general population)	Both, >18 y	PRAL, NEAP	All mentioned assessment tools	No significant association between total cholesterol, LDL-C, HDL-C and dietary PRAL, as well as NEAP. No significant association between TG and dietary NEAP.

**Abbreviations**: 24 HR, 24-h dietary recall questionnaire; A:P, animal-protein-to-potassium ratio; BDHQ, brief validated self-administered diet history questionnaire; TAG, triacylglycerol; LDL-C, low-density lipoprotein; HDL-C, high-density lipoprotein; TG, triglyceride; HR, hazard ratio; NEAP, net-endogenous acid production; PRAL, potential renal acid load; FD, food diary; FFQ, food frequency questionnaire; NAE, urine net acid excretion; BMI, body mass index; T2DM, type 2 diabetes mellitus; DAL, dietary acid load; WMD, weighted mean difference; CVD, cardiovascular disease. * Indicates consecutive outcomes that stemmed from one study, but differences in the significance of association between lipid metabolism values and DAL were obtained.
